# Supplementary material for: Identification and implication of tissue-enriched ligands in epithelial–endothelial crosstalk during pancreas development
Source: Sci Rep. 2022 Jul 21;12:12498. doi: 10.1038/s41598-022-16072-y (PMC9304391; doi:10.1038/s41598-022-16072-y)
Supplement: Supplementary file 1 — Supplementary Information. [file 41598_2022_16072_MOESM1_ESM.docx]

| **­­Cell types** | **Markers genes** | **References** |
| --- | --- | --- |
| Epithelial general | Cdh1 | ^1^ |
| Epithelial general | Cldn6 | ^2^ |
| Epithelial ductal | Spp1 | ^3^ |
| Epithelial ductal | Sox9 | ^4^ |
| Epithelial acinar | Ptf1a | ^5^ |
| Epithelial acinar | Amy2b | ^6^ |
| Epithelial endocrine | Ins1 | ^6^ |
| Epithelial endocrine | Gcg | ^6^ |
| Epithelial endocrine | Pax4 | ^7^ |
| Epithelial endocrine | Insm1 | ^8^ |
| Mesenchyme general | Col1a1 | ^9^ |
| Mesothelial | Wt1 | ^10^ |
| Mesothelial | Upk3b | ^11^ |
| Mesenchyme subpopulation | Rgs5 | ^12^ |
| Mesenchyme subpopulation | Des | ^13^ |
| Neurons | Dlx2 | ^14^ |
| Neurons | Syt1 | ^15^ |
| Immune | Itgam | ^16^ |
| Immune | Cd53 | ^17^ |
| Endothelial | Cdh5 | ^18^ |
| Endothelial | Kdr | ^19^ |
| Erythrocyte | Gypa | ^20^ |
| Erythrocyte | Slc4a1 | ^21^ |
| Tip | Dll4 | ^22^ |
| Tip | Cd34 | ^23^ |
| Arterial | Sox17 | ^24^ |
| Arterial | Gja5 | ^25^ |
| Arterial | Efnb2 | ^26^ |
| Stalk | Vwf | ^27^ |
| Stalk | Cd36 | ^28^ |
| Venous | Nr2f2 | ^29^ |
| Venous | Emcn | ^30^ |
| Venous | Ephb4 | ^31^ |
| Lymphatic | Prox1 | ^32^ |
| Lymphatic | Flt4 | ^33^ |

**Table S1: List of cell population marker genes**

**References of Table S1**

1. An, J., Zheng, Y. & Dann, C. T. Mesenchymal to Epithelial Transition Mediated by CDH1 Promotes Spontaneous Reprogramming of Male Germline Stem Cells to Pluripotency. *Stem Cell Rep.* **8**, 446–459 (2017).

2. Turksen, K. & Troy, T.-C. Claudin-6: A novel tight junction molecule is developmentally regulated in mouse embryonic epithelium. *Dev. Dyn.* **222**, 292–300 (2001).

3. Scavuzzo, M. A. *et al.* Endocrine lineage biases arise in temporally distinct endocrine progenitors during pancreatic morphogenesis. *Nat. Commun.* **9**, 3356 (2018).

4. Seymour, P. A. Sox9: A Master Regulator of the Pancreatic Program. *Rev. Diabet. Stud.* **11**, (2014).

5. Jakubison, B. L. *et al.* Induced PTF1a expression in pancreatic ductal adenocarcinoma cells activates acinar gene networks, reduces tumorigenic properties, and sensitizes cells to gemcitabine treatment. *Mol. Oncol.* **12**, 1104–1124 (2018).

6. Qadir, M. M. F. *et al.* Single-cell resolution analysis of the human pancreatic ductal progenitor cell niche. *Proc. Natl. Acad. Sci.* **117**, 10876–10887 (2020).

7. Napolitano, T. *et al.* Pax4 acts as a key player in pancreas development and plasticity. *Semin. Cell Dev. Biol.* **44**, 107–114 (2015).

8. Osipovich, A. B. *et al.* Insm1 promotes endocrine cell differentiation by modulating the expression of a network of genes that includes Neurog3 and Ripply3. *Development* **141**, 2939–2949 (2014).

9. Millington-Ward, S. *et al.* RNAi of COL1A1 in mesenchymal progenitor cells. *Eur. J. Hum. Genet.* **12**, 864–866 (2004).

10. Carneiro, F. P. *et al.* A panel of markers for identification of malignant and non-malignant cells in culture from effusions. *Oncol. Rep.* **38**, 3538–3544 (2017).

11. Kanamori-Katayama, M. *et al.* LRRN4 and UPK3B Are Markers of Primary Mesothelial Cells. *PLOS ONE* **6**, e25391 (2011).

12. Hu, M. *et al.* Over-expression of regulator of G protein signaling 5 promotes tumor metastasis by inducing epithelial–mesenchymal transition in hepatocellular carcinoma cells. *J. Surg. Oncol.* **108**, 192–196 (2013).

13. Liu, Y., Deng, B., Zhao, Y., Xie, S. & Nie, R. Differentiated markers in undifferentiated cells: Expression of smooth muscle contractile proteins in multipotent bone marrow mesenchymal stem cells. *Dev. Growth Differ.* **55**, 591–605 (2013).

14. Petryniak, M. A., Potter, G. B., Rowitch, D. H. & Rubenstein, J. L. R. Dlx1 and Dlx2 Control Neuronal versus Oligodendroglial Cell Fate Acquisition in the Developing Forebrain. *Neuron* **55**, 417–433 (2007).

15. Tarquis-Medina, M. *et al.* Synaptotagmin-13 Is a Neuroendocrine Marker in Brain, Intestine and Pancreas. *Int. J. Mol. Sci.* **22**, 12526 (2021).

16. Solovjov, D. A., Pluskota, E. & Plow, E. F. Distinct Roles for the α and β Subunits in the Functions of Integrin αMβ2*. *J. Biol. Chem.* **280**, 1336–1345 (2005).

17. Hořejši, V. & Vlček, Č. Novel structurally distinct family of leucocyte surface glycoproteins including CD9, CD37, CD53 and CD63. *FEBS Lett.* **288**, 1–4 (1991).

18. Feng, W., Chen, L., Nguyen, P. K., Wu, S. M. & Li, G. Single Cell Analysis of Endothelial Cells Identified Organ-Specific Molecular Signatures and Heart-Specific Cell Populations and Molecular Features. *Front. Cardiovasc. Med.* **6**, (2019).

19. Terman, B. I. *et al.* Identification of the KDR tyrosine kinase as a receptor for vascular endothelial cell growth factor. *Biochem. Biophys. Res. Commun.* **187**, 1579–1586 (1992).

20. Cartron, J.-P. & Rahuel, C. Human Erythrocyte Glycophorins: Protein and Gene Structure Analyses. *Transfus. Med. Rev.* **6**, 63–92 (1992).

21. Kuo, M.-S. *et al.* Different Involvement of Band 3 in Red Cell Deformability and Osmotic Fragility—A Comparative GP.Mur Erythrocyte Study. *Cells* **10**, 3369 (2021).

22. The endothelial tip-stalk cell selection and shuffling during angiogenesis | SpringerLink. https://link.springer.com/article/10.1007/s12079-019-00511-z.

23. Siemerink, M. J. *et al.* CD34 marks angiogenic tip cells in human vascular endothelial cell cultures. *Angiogenesis* **15**, 151–163 (2012).

24. Corada, M. *et al.* Sox17 is indispensable for acquisition and maintenance of arterial identity. *Nat. Commun.* **4**, 2609 (2013).

25. Buschmann, I. *et al.* Pulsatile shear and Gja5 modulate arterial identity and remodeling events during flow-driven arteriogenesis. *Development* **137**, 2187–2196 (2010).

26. Wolf, K., Hu, H., Isaji, T. & Dardik, A. Molecular identity of arteries, veins, and lymphatics. *J. Vasc. Surg.* **69**, 253–262 (2019).

27. Chen, W. *et al.* The endothelial tip-stalk cell selection and shuffling during angiogenesis. *J. Cell Commun. Signal.* **13**, 291–301 (2019).

28. Chen, W. *et al.* The endothelial tip-stalk cell selection and shuffling during angiogenesis. *J. Cell Commun. Signal.* **13**, 291–301 (2019).

29. Cui, X. *et al.* Venous Endothelial Marker COUP-TFII Regulates the Distinct Pathologic Potentials of Adult Arteries and Veins. *Sci. Rep.* **5**, 16193 (2015).

30. Park-Windhol, C. *et al.* Endomucin inhibits VEGF-induced endothelial cell migration, growth, and morphogenesis by modulating VEGFR2 signaling. *Sci. Rep.* **7**, 17138 (2017).

31. Wolf, K., Hu, H., Isaji, T. & Dardik, A. Molecular identity of arteries, veins, and lymphatics. *J. Vasc. Surg.* **69**, 253–262 (2019).

32. Wilting, J. *et al.* The transcription factor Prox1 is a marker for lymphatic endothelial cells in normal and diseased human tissues. *FASEB J.* **16**, 1271–1273 (2002).

33. Kong, L.-L. *et al.* The optimum marker for the detection of lymphatic vessels (Review). *Mol. Clin. Oncol.* **7**, 515–520 (2017).

**Table S2: Number of detected potential interactions between the sender and target populations**

|  |  | Target populations | | | | | |
| --- | --- | --- | --- | --- | --- | --- | --- |
|  |  | Epithelial | Mesenchyme | Endothelial | Immune | Neurons | Erythrocyte |
| Sender populations | Epithelial | 1582 | 1659 | 1730 | 1628 | 1567 | 1603 |
|  | Mesenchyme | 2470 | 2614 | 2739 | 2585 | 2487 | 2511 |
|  | Endothelial | 533 | 571 | 589 | 563 | 536 | 542 |
|  | Immune | 1387 | 1440 | 1572 | 1551 | 1376 | 1413 |
|  | Neurons | 547 | 568 | 594 | 555 | 542 | 553 |
|  | Erythrocyte | 0 | 0 | 0 | 0 | 0 | 0 |

**Figure S1**

**
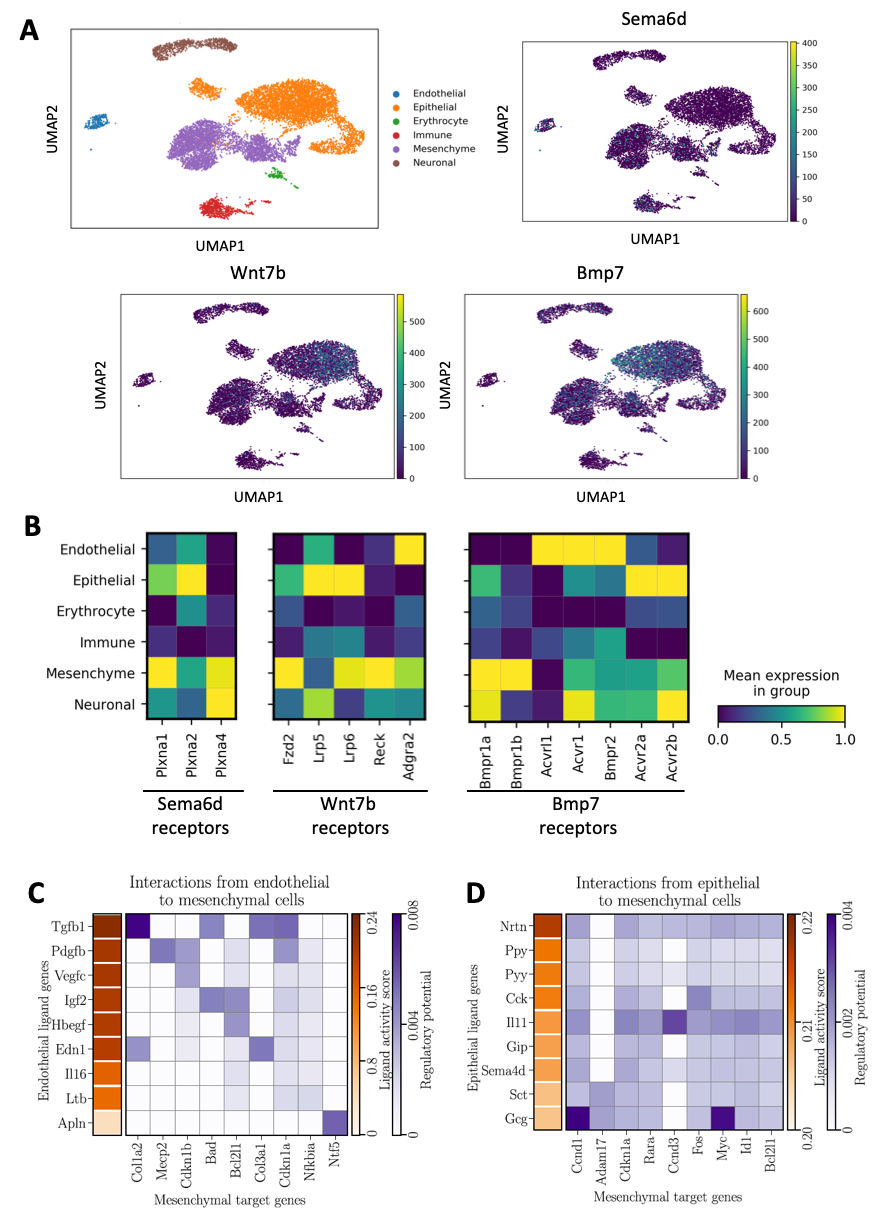
**

**Figure S1: Expression of the selected ligands identified by NicheNet and their receptors**. **(A)** UMAP plots of the expression level of the selected endothelial and epithelial ligands identified by NicheNet. **(B)** Expression profiles of the receptors for the ligands selected. **(C-D)** NicheNet’s interactomic predictions from endothelial to mesenchymal (C) and from epithelial to mesenchymal (D). The ligands are ranked based on their activity scores (orange color map) while their regulation potential on target genes are colored in violet.

**Figure S2**

**
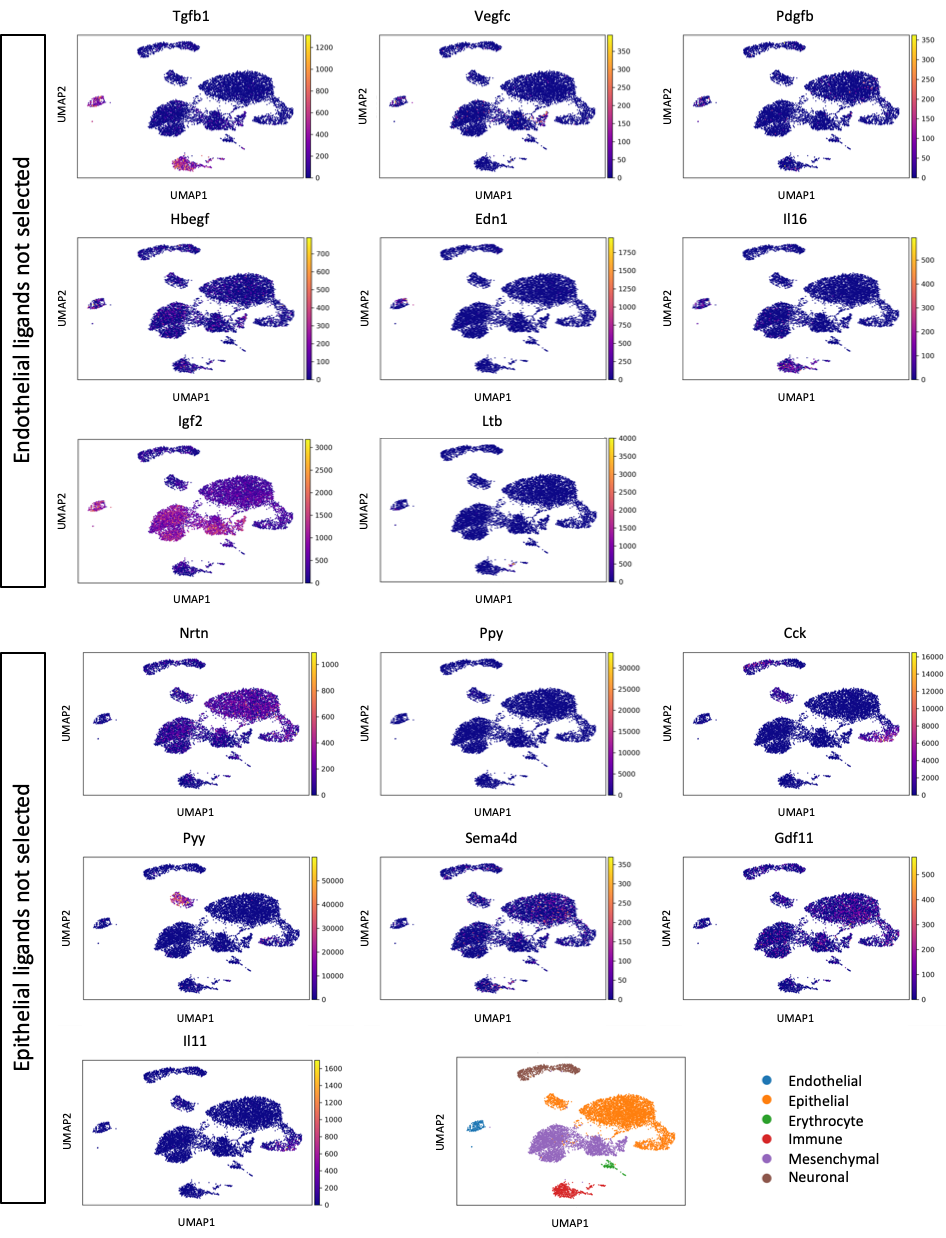
**

**Figure S2: UMAP plots showing the expression levels of endothelial and epithelial ligands identified by NicheNet, but not selected for further experimental analyses.**

**Figure S3**

**
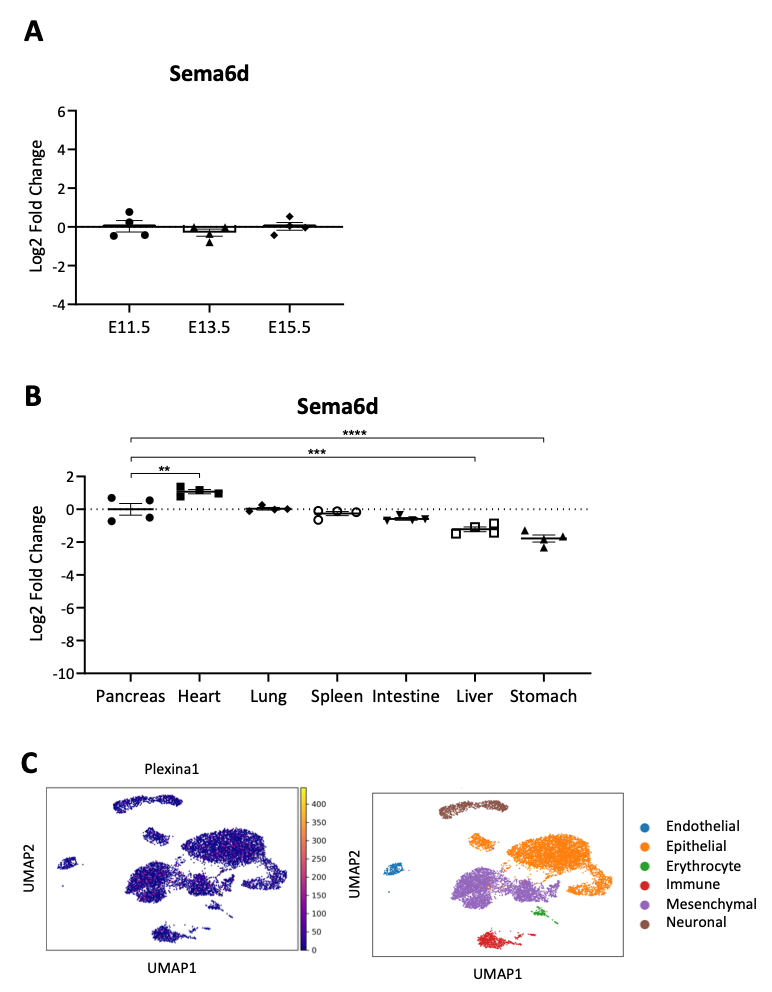
**

**Figure S3: Expression of Sema6d during pancreas organogenesis and in different organs. (A)** RT-qPCR analysis of Sema6d expression at three stages (E11.5 - E13.5 - E15.5) of pancreas development (n=4). Sema6d expression was stable from E11.5 to E15.5. **(B)** Comparative analysis of the expression of Sema6d by RT-qPCR in the pancreas and other organs at E15.5 (n=4), reveals similar expression levels. Actb and Rpl27 were used as housekeeping genes (ΔCt) and results are presented as log2 fold change (log2 (2-ΔΔCt)), as compared to E11.5 (A) or to pancreatic tissue (B). ** p<0.005, *** p<0.0005, **** p<0.0001. **(C)** UMAP plot of the expression level of PlexinA1 in the different cell populations.

**A**

**Figure S4**

**Figure S4: Expression of Wnt7b and BMP7 during pancreas organogenesis and in different organs. (A)** RT-qPCR analysis of Cdh1, Wnt7b and Bmp7 at three stages (E11.5 - E13.5 - E15.5) of pancreas development (n=4). Increased expression of Cdh1 from E11.5 to E15.5, explains the decreased expression of Wnt7b and Bmp7 upon normalization on Cdh1 (right graphs). Comparative analysis of the expression of Wnt7b **(B)** and Bmp7 **(C)** by RT-qPCR in the pancreas and other organs at E15.5 (n=4), shows higher expression of these two transcripts in the pancreas. Actb and Rpl27 were used as housekeeping genes (ΔCt) and results are presented in log2 fold change (log2 (2-ΔΔCt)), as compared to E11.5 (A) or to pancreatic tissue (B,C). * p<0.05, ** p<0.005, *** p<0.0005, **** p<0.0001.

**Figure S5**

**Figure S5: Bmp7 can directly signal to endothelial cells without severely affecting the epithelial compartment. (A)** Immunolabeling of phospho Smad 1/5+ (red), epithelial E-Cad+ (white) and endothelial VE-Cad+ (green) cells, with Hoechst nuclei counterstaining (blue) on pancreatic explants treated with BMP7 for 90 minutes (BMP7, 400 ng/mL), or left untreated (CTL). Regions delineated by dashed lines are magnified below, and illustrate increased phospho Smad 1/5 positive cells (endothelial and non-endothelial) in BMP7-treated explants. Scale bar : 100 μm and 10 μm (magnification). **(B)** RT-qPCR analysis of Bmp target genes Id1, Id2 and Id3 in primary endothelial cells, normalized to Actb and Rpl27, and presented in log2 fold change, in BMP7 (400 ng/mL) or DMH-1 (3 µM) treated cells, as compared to untreated control cells cultured for 48h (n=3-7). Expression of Id genes was upregulated with BMP7 and downregulated with DMH-1. **(C)** RT-qPCR analysis of general (Cdh1), tip (Amy2a and Ptf1a) and trunk (Krt19 and Sox9) epithelial markers normalized to Actb and Rpl27, and presented as log2 fold change, in BMP7- and DMH-1-treated explants as compared to controls cultured for 48h (n=6-9). No significant changes were measured, but a trend to an increase of tip markers was noticed. **(D)** Immunolabeling of amylase+ (red), epithelial E-Cad+ (white) and endothelial VE-Cad+ (green) cells, with Hoechst nuclei counterstaining (blue). Region delineated by dashed lines is magnified below. Quantification of the amylase+ surface reported to total Hoechst+ surface of control, BMP7- and DMH-1-treated explants cultured for 72h (n=6), revealed a slight but not significant increase of the acinar surface in BMP7 cultures. Scale bars: 100 μm and 10 μm (magnification). One-way ANOVA (comparison to CTL): * p<0.05, ** p<0.005.
